# A Longitudinal RCT on the Effectiveness of a Psychological Intervention for Hospital Healthcare Workers During the COVID-19 Pandemic: What We Learned to Date

**DOI:** 10.1007/s10880-023-09988-8

**Published:** 2024-01-31

**Authors:** Damiano Rizzi, Michela Monaci, Giulia Gambini, Ilaria Maria Antonietta Benzi, Stefano Perlini, Annalisa De Silvestri, Catherine Klersy, Lavinia Barone

**Affiliations:** 1 Fondazione Soleterre Strategie di Pace ONLUS, Milan, Italy; 2 IRCCS Policlinico San Matteo Foundation, University of Pavia, Pavia, Italy; 3 Department of Brain and Behavioral Sciences, University of Pavia, Piazza Botta Adorno Antoniotto, 11, 27100 Pavia, Italy

**Keywords:** COVID-19 pandemic, Healthcare workers, DBT-informed psychological intervention, RCT longitudinal study

## Abstract

The COVID-19 pandemic has led to significant psychological distress among frontline healthcare workers (HCWs), with a particular increase in trauma-related symptoms. This study investigated the longitudinal course of trauma-associated symptoms and behaviors in HCWs and the effectiveness of a brief dialectical behavior therapy (DBT)-informed intervention in mitigating these symptoms over 12 months. The trial included 225 HCWs randomly assigned to one of three groups: no intervention (control), in-person DBT-informed intervention, or online DBT-informed intervention. Over time, a natural decrease in PTSD symptoms was observed in all groups. Contrary to expectations, no difference was found between the control and intervention groups. However, for participants with severe PTSD symptoms, the intervention significantly mitigated their distress. No differences emerged between in-person and online interventions, suggesting equal effectiveness. Females reported higher trauma-related symptoms, while no differences emerged among different professional roles. These findings underscore the importance of targeted interventions for HCWs experiencing severe symptoms and highlight the potential of online modalities. Further research is needed to optimize the deployment of mental health resources within the healthcare setting, particularly during crises.

## Introduction

Throughout the COVID-19 pandemic, healthcare systems and healthcare professionals confronted several challenges. Indeed, many healthcare workers (HCWs) contracted the virus, resulting in numerous cases and fatalities (Amerio et al., 2020). Moreover, hospital units underwent major transformations (Marcon et al., 2020), with intensive care units being extensively restructured to accommodate the increasing volume and numbers of COVID-19 patients (Bersano et al., 2020). Confronted by various difficulties, such as an overwhelming workload, inadequate protective equipment, and the initial lack of specific treatment protocols, HCWs emerged as a particularly susceptible group to psychological distress during the pandemic (Walton et al., 2020). The risk was particularly heightened for frontline HCWs, who were directly engaged with COVID-19 patients, leading to an elevated incidence of trauma-related symptoms and adverse mental health outcomes (Lai et al., 2020). Indeed, numerous studies conducted during the COVID-19 pandemic have indicated a high prevalence of psychological disorders among healthcare workers (HCWs). For instance, Pappa et al. (2020) reported a pooled prevalence of 23.2% for anxiety, 22.8% for depression, and 38.9% for insomnia among HCWs (Pappa et al., 2020). Similar findings were echoed in a meta-analysis by Luo et al. (2020), which included 62 studies (*N* = 162,639) from 17 countries, indicating pooled prevalences of 26%, 25%, and 32% for anxiety, depression, and insomnia, respectively (Luo et al., 2020). Furthermore, Vizheh et al. (2020) identified high levels of psychological distress, with a prevalence of anxiety, depression, and stress ranging from 24.1% to 67.6%, 12.1% to 55.9%, and 29.8% to 63.0%, respectively (Vizheh et al., 2020).

The psychological functioning of frontline healthcare workers (HCWs), particularly concerning trauma-related symptoms and behaviors, merits scrutiny. Posttraumatic stress disorder (PTSD), a mental disorder featured by four symptom clusters—re-experiencing, avoidance, negative cognition, and mood, and heightened arousal and reactivity following exposure to traumatic events (APA, 2013), can persist long after natural disasters, such as pandemics. For example, Lee et al. (2007) discovered that HCWs who survived the 2003 SARS outbreak exhibited elevated stress levels and more pronounced PTSD symptoms one year post-pandemic compared to non-HCWs (Lee et al., 2007). Another study revealed that HCWs were significantly more susceptible to PTSD even three years post-pandemic, with a 40% persistence rate. This was particularly prevalent among those who served in emergency units, were subjected to quarantine, or had relatives infected with SARS (Wu et al., 2009).

Likewise, the COVID-19 pandemic presented heightened risks for HCWs to experience trauma-related symptoms, primarily due to specific factors such as the rapid surge in severely ill patients, high mortality rates, and other occupational stressors (Carmassi et al., 2020). Indeed, in this context, HCWs found themselves directly exposed to traumatic events intrinsic to their roles, such as confronting emergencies in frontline units and consistently interfacing with patient illnesses and mortality (Mosheva et al., 2021). A recent study conducted in an intensive care unit reported that 40% of medical staff exhibited severe PTSD symptoms (Greenberg et al., 2021). Further, a meta-analysis by d'Ettorre et al. (2021) revealed the prevalence of PTSD among HCWs to be between 2.1% and 73.4%, with the highest rates coinciding with the peak period of the COVID-19 pandemic and care provision for COVID-19 patients in emergency wards (d'Ettorre et al., 2021). Another study found that trauma-related symptoms were higher among women and professionals with low social support during the COVID-19 pandemic and during the SARS and MERS pandemics, primarily due to the self-isolation and stigmatization of HCWs as potential infection carriers (Carmassi et al., 2020). Italy faced one of the most challenging situations, being at the forefront of the pandemic with approximately 200,000 deaths. As the second country to confront a significant spread of COVID-19 cases following China, Italy witnessed a marked and escalating deterioration in the psychological conditions of its HCWs (Rizzi et al., 2022).

Another crucial consideration pertains to the permanence versus transience of the reported impairments in psychological functioning. Again, the evidence presents a diverse picture. On the one hand, some longitudinal studies have reported decreased psychological and trauma-related symptoms over time (Caramello et al., 2023; Robinson et al., 2022). Conversely, other research has underscored the potential for some psychological issues, like depression and PTSD, to persist and even extend beyond the duration of the pandemic, thereby necessitating prolonged vigilance and monitoring (Giusti et al., 2020; Taylor, 2019). On the whole, the COVID-19 pandemic represented a formidable challenge for HCWs, regardless of their role (i.e., medical doctors, nurses, or social-health workers), who were exposed to the suffering of patients (Ruiz-Fernández et al., 2020), required to manage extended shifts and abrupt changes in their work routines (Galli et al., 2020), compelled to make critical ethical decisions in the face of resource scarcity (Greenberg et al., 2021), and confronted with death, anxiety, and fear of infection (Cai et al., 2020).

Given this backdrop, studies underscored the pressing need to implement psychological interventions, both in person and online, for HCWs to mitigate adverse mental health outcomes and safeguard their caregiving capabilities intrinsic to their professions (Chirico et al., 2020; Orrù et al., 2020; Parolin et al., 2021; Vizheh et al., 2020). Yet, despite some work contributing to the analysis of the effectiveness of these interventions (Varela et al., 2023), the body of literature on successful strategies to alleviate psychological distress and PTSD in HCWs during the COVID-19 pandemic remains limited.

Furthermore, systematic evaluations of existing and long-term mental health management strategies are lacking (Bertuzzi et al., 2021; De Kock et al., 2021). Although the importance and quantity of trauma-focused interventions for PTSD are on the rise, data have yet to demonstrate the expected efficacy consistently. A recent network meta-analysis on RCT psychological interventions with patients with a primary diagnosis of PTSD considered the relative effectiveness of a range of psychotherapies modalities (i.e., 82 RCT trials including 5838 patients in the time range 1991–2020), did not find any highly divergent levels of efficacy and acceptability among individual face-to-face interventions (Jericho et al., 2022).

Reliable and sustainable psychological interventions in community settings, such as hospital units primarily engaged in the COVID-19 pandemic, confront at least two main research questions that have only been partially addressed. The first pertains to the possible high incidence of psychological distress in HCWs during the pandemic and whether this distress is transient or permanent. The second relates to the effectiveness of sustainable and reliable interventions designed to decrease posttraumatic psychological conditions in all professional roles of HCWs and promote their well-being.

Further research is needed for a more accurate and reliable exploration of the severity and longitudinal trajectories of the psychological symptoms, precisely trauma-related symptoms and behaviors, and the short/long-term effects of psychological interventions targeting these symptoms (Varela et al., 2023).

### Aims of the Study

The main aims of this study were: (1) to explore the longitudinal course of trauma-associated symptoms and behaviors in frontline healthcare workers during and after the second waves of the COVID-19 pandemic, (2) to longitudinally assess the efficacy of a psychological intervention versus non-intervention condition on trauma-related symptoms and behaviors aimed at healthcare workers, accounting for (3) gender and professional role differences in these pathways.

Thus, in line with the available literature, we hypothesized the following. First, healthcare workers will show a decline in the severity of trauma-related symptoms over time. Second, the psychological intervention would significantly decrease HCWs' trauma-related symptoms at T1 (i.e., six months after the intervention) and the 12-month follow-up (T2) compared to no intervention. Notably, we expected the psychological intervention to effectively lower the severity of trauma-related symptoms in the most psychologically distressed HCWs (i.e., individuals with moderate to severe symptoms) at T1 (i.e., six months after the intervention) and at the 12-month follow-up (T2) compared to no intervention, with in-person intervention has shown to be more effective than online intervention. Moreover, we expected to find significant gender and professional role differences in these longitudinal courses.

## Material and Methods

### Study Design and Procedures

This is an Italian randomized controlled trial to evaluate the efficacy of a psychological intervention on the traumatic effects of COVID-19 on healthcare workers' mental health at the Fondazione IRCCS Policlinico San Matteo in Pavia (Italy).

Inclusion criteria for participation were as follows: (1) being at least 18 years old; (2) working at the Fondazione IRCCS Policlinico San Matteo as a medical doctor, nurse, or social-health worker (OSS); and (3) being on duty as a frontline healthcare worker in one of the frontline units of the Hospital during the COVID-19 emergency (Infectious disease unit, Pneumology, Emergency Department, Intensive Care Unit). All participants provided informed consent. They were informed of the study's duration, potential risks and benefits, and data protection and privacy details. Additionally, after the intervention, participants in the control group were offered access to a psychological support group.

**Fig. 1:**
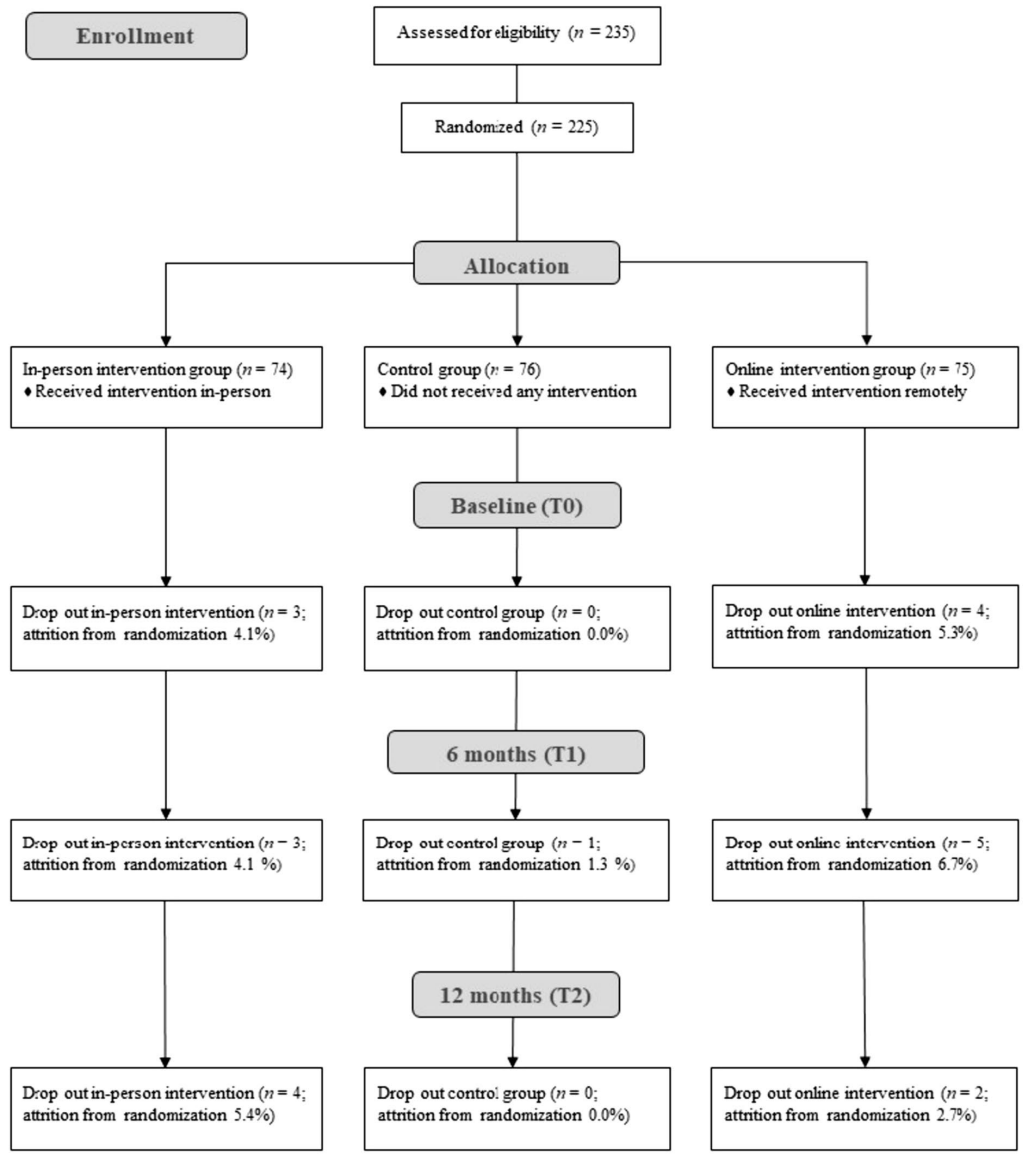
Participants’ flowchart through the study from screening to post-intervention data collection

Participants were assigned a unique reference code and provided with self-report questionnaires for paper/pencil administration to protect their anonymity. We randomly assigned participants to three conditions: control group, in-person intervention group, and online intervention group. Data collection began in February 2021 and ended in October 2022, including three time points: pre-intervention (T0), six months post-intervention (T1), and 12-month follow-up (T2). See Fig. 1 for a comprehensive overview of the data collection process.

The study adhered to APA ethical standards, the Declaration of Helsinki, and the Ethical Committee of the Fondazione IRCCS Policlinico San Matteo approved all materials and procedures.

### Participants

Participants were randomized in a 1:1:1 ratio using a variable-size block list, which was generated before the beginning of the study through the 'ralloc' command in the STATA statistical software. 225 individuals were recruited using a simple random sampling method without replacement. The average age across all groups was 44 years. Males represented 42% of the total sample. Smoking habits indicated that the majority were non-smokers (57% overall). Regarding marital status, 71% were married or in a civil union. Half of the participants reported having offspring, and of those, 47% had one child. The professional roles of participants were categorized as medical doctors, nurses, or social-health workers, with the sample comprising 40% physicians, 40% nurses, and 20% other healthcare workers. Most participants worked in the Intensive Care department (42% overall), followed by Infectious Diseases (17%), Pneumology (13%), and the Emergency Department (28%). Regarding years in their occupation, 32% had been in their roles for 10–20 years, 29% had more than 20 years of experience, 20% had worked for 1–5 years, and 19% had been in their profession for 5–10 years. See Table 1 for detailed demographics.

**Table 1:** Overview of the DBT-informed intervention structured format

Session	Focus of the session
Session 1	Orientation and goals definition
	Mindfulness practice: grounding (observe and describe your breath)
	Defining goals and targets of the client by using validation (levels 1, 2, 3)
	Closing session Mindfulness practice (paced breathing)
Session 2	Mindfulness practice: grounding (observe and describe your breath)
	Defining goals and targets of the client by using validation (levels 1, 2, 3, 4)
	Teaching and observing states of mind (emotional mind, rational mind, wise mind) by discussing trauma-related events
	Closing session Mindfulness practice on wise mind search
Session 3	Mindfulness practice: observe and describe your thoughts
	Observation and validation of client’s thoughts
	Identification of a target trauma-related behavior
	Chain analysis and problem-solving
	Closing session mindfulness practice
Session 4	Mindfulness practice: observe and describe your thoughts
	Observation and validation of client’s thoughts
	Behaviors that require radical acceptance
	Identification of a target behavior
	Chain analysis and problem-solving
	Closing session mindfulness practice
Session 5	Mindfulness practice: observe and describe your thoughts
	Observation and validation of client’s thoughts
	Behaviors that require radical acceptance
	Identification of a target behavior
	Chain analysis and problem-solving
	Commitment strategies for keeping intervention achievements and mindfulness practice over time
	Closing session mindfulness practice

The brief intervention of five 50-min individual sessions, delivered to all subject belonging to the in-person intervention group or to the online intervention group (Linehan et al., 1993; Linehan & Wilks, 2015)

All available frontline workers were recruited for the study. We applied a top-down approach, starting with comparing the intervention vs. no intervention cohorts; in case of significant difference, in-person and remote groups were compared. With 64 subjects in the control arm and 128 subjects (64 in-person, 64 online) in the intervention arm, we could elicit an effect of 0.5 standard deviations with power over 90% and a 2-sided type I error of 5%. To account for dropout, 75 subjects per arm were enrolled.

### Psychological Intervention

The psychological intervention was a brief version of a dialectical behavior therapy (DBT)-informed intervention program. DBT, developed by Linehan (Linehan, 1993; Linehan & Wilks 2015), is a treatment that utilizes various cognitive and behavioral techniques guided by a dialectical approach. There is extensive support for DBT's efficacy with borderline personality disorder patients and several comorbid clinical conditions by decreasing psychiatric symptoms related to psychological distress and increasing emotional regulation skills and quality of life (Harvey et al., 2019; Neacsiu et al., 2014; Storebø et al., 2020). The focus of the treatment is emotional regulation, which is the core of the trauma-related symptoms and behaviors inquired. Therefore, DBT implies a multimodal approach, including core strategies (i.e., validation and problem-solving) and specific skills to be acquired by the patient (i.e., mindfulness, distress tolerance, emotion regulation, and interpersonal effectiveness).

The brief DBT-informed intervention was designed by a certified DBT therapist and trainer, Lavinia Barone, who trained a team of psychologists from the Soleterre Foundation in the short intervention program during a tailored 20 h course and then monitored their clinical work with periodic supervisions. The Soleterre Foundation carefully chose the psychologist responsible for the intervention. In addition, this non-governmental organization (NGO) has established both in-person and online psychological support services within the Fondazione IRCCS Policlinico San Matteo.

The brief intervention was designed to reduce trauma-related behaviors and symptoms in individuals: it consisted of five 50-min individual sessions, delivered either in-person or online in real time (synchronously) to the two intervention groups, each session following a structured format*.* The sessions were centered around dialectical behavior therapy (DBT) principles and techniques and focused on fostering exposure to painful events, distress tolerance, and emotional regulation through active exercises. The exercises incorporated various DBT techniques such as mindfulness, validation, chain analysis problem-solving, non-judgmental stance, and radical acceptance. In addition, this approach aimed to enhance participants' emotional self-compassion, awareness, and coping skills, which are crucial for mental health and well-being (see Table 2 for an in-depth overview of the structured format). Moreover, participants were provided with printed brochures and leaflets on adaptive behaviors during emergencies. Also, information were available in audio format.

**Table 2:** Study participants' demographics in the three randomized groups (Control, Online intervention, In-person intervention) and the Intervention group

Variables	No intervention (Control) (*n* = 76)	Online (*n* = 75)	In-person (*n* = 74)	Total sample (*n* = 225)
	*M* (SD)	*M* (SD)	*M* (SD)	*M* (SD)
Age (years)	44 (11)	43 (10)	44 (10)	44 (11)
	*n* (%)	*n* (%)	*n* (%)	*n* (%)
Male	35 (46)	29 (39)	31 (42)	95 (42)
Smoker				
Yes	26 (34)	20 (27)	19 (26)	65 (29)
No	38 (50)	44 (59)	46 (62)	128 (57)
Ex	10 (13)	9 (12)	7 (9)	26 (12)
Electronic cigarette	2 (3)	2 (3)	2 (3)	6 (3)
Status				
Single/unmarried	22 (29)	9 (12)	14 (19)	45 (20)
Married/civil union/married	48 (63)	59 (79)	53 (72)	160 (71)
Widowed	1 (1)	1 (1)	1 (1)	3 (1)
Separated/divorced	5 (7)	6 (8)	6 (8)	17 (8)
Living situation/habitual cohabitation for the past three years				
With partner and children	35 (46)	33 (44)	30 (40)	98 (44)
With partner only	16 (21)	28 (37)	28 (38)	72 (32)
With children only	1 (1)	2 (3)	1 (1)	4 (2)
With parents	4 (5)	4 (5)	1 (1)	9 (4)
With other family members	1 (1)	1 (1)	0 (0)	2 (1)
With friends	6 (8)	0 (0)	0 (0)	6 (3)
Alone	13 (17)	7 (9)	14 (19)	34 (15)
Have offsprings	38 (50)	39 (52)	35 (47)	112 (50)
Number of offsprings				
1	20 (53)	18 (47)	14 (40)	52 (47)
2	16 (42)	16 (42)	16 (33)	48 (43)
More than two	2 (5)	4 (11)	5 (15)	11 (10)
Change in child management after COVID-19	13 (34)	15 (40)	15 (43)	43 (39)
Living parents	69 (91)	63 (85)	63 (85)	195 (87)
Parents need assistance	20 (29)	13 (20)	11 (18)	44 (23)
Change in parents' management after COVID-19	12 (60)	2 (15)	7 (64)	21 (48)
Satisfied with parents' situation	49 (71)	53 (84)	52 (83)	154 (79)
Living parents-in-law	43 (80)	55 (85)	48 (80)	146 (82)
Parents-in-law need assistance	9 (21)	7 (12)	5 (10)	21 (15)
Change in parents-in-law management after COVID-19	6 (67)	2 (29)	1 (20)	9 (43)
Satisfied with parents-in-law situation	37 (86)	50 (91)	46 (96)	133 (91)
Occupation				
Doctor	31 (41)	29 (39)	30 (41)	90 (40)
Nurse	33 (43)	30 (40)	29 (39)	92 (41)
Social-health worker	12 (16)	16 (21)	15 (20)	43 (19)
Current department				
Intensive care	31 (41)	33 (44)	31 (42)	95 (42)
Infectious diseases	11 (14)	10 (13)	17 (23)	38 (17)
Pneumology	9 (12)	11 (15)	10 (13)	30 (13)
Emergency department	25 (33)	21 (28)	16 (26)	62 (28)
Years of occupation				
1–5 years	13 (17)	17 (23)	14 (19)	44 (20)
5–10 years	13 (17)	11 (15)	19 (26)	43 (19)
10–20 years	25 (33)	27 (36)	20 (27)	72 (32)
− > 20 years	25 (33)	20 (27)	21 (28)	66 (29)
Change occupation during COVID-19	12 (16)	14 (18)	15 (20)	41 (18)
Satisfied with occupation	50 (66)	51 (68)	50 (68)	151 (67)
Psychological Intervention before COVID-19	21 (28)	19 (26)	18 (25)	58 (33)
Psychological Intervention during COVID-19	11 (15)	10 (13)	9 (12)	30 (13)

Data are presented as the mean and standard deviation of continuous and as count and percentile if categorical

### Measures

The *National Stressful Events Survey PTSD Short Scale* (NSESSS) (Kilpatrick et al., 2013) is a 9-item self-report that measures the severity of posttraumatic stress symptoms. Respondents rate how much they were bothered by each symptom during the past week on a 5-point scale from 0 (not at all) to 4 (extremely). The total score ranges from 0 to 36, and this score is used to assess the severity of PTSD symptoms. Alternatively, the average total score can be calculated by dividing the total score by the number of items, which can easily categorize the overall severity of PTSD into five levels: none (0), mild (1), moderate (2), severe (3), and extreme (4). Cronbach's alpha for the scale showed good internal consistency between measurements (range *α*: 0.84–0.89).

### Statistical Analysis

We used Stata 17 (StataCorp, College Station, TX, USA) for computation. The confidence intervals of the estimates were calculated at the 95% level. All statistical tests were 2-tailed, and a *p* < 0.05 was considered statistically significant. All subjects that completed baseline data gathering were included in the analyses, accounting for the intention-to-treat principle (McCoy, 2017). This approach ensures that our analysis remains unbiased, reflecting the potential real-world scenarios where not all individuals who start an intervention will necessarily complete it.

We described continuous variables with means, standard deviations (SD), and categorical variables as counts and percentiles. The comparison groups were the untreated subjects and those receiving psychological support, including online and in-person interventions. In case of significant differences in the NSESSS analysis, comparisons between all three arms were planned.

The analyses for the study objectives were performed using regression models for repeated measures; Huber-White robust standard errors were computed to account for the intra-subject correlation of measurements. We fitted both within-group models to assess changes over time and between groups models to determine the difference in change over time; the test for interaction between the treatment group and time was used to assess the difference between groups. We also performed a predefined subgroup analysis based on the baseline severity of the NSESSS score (</≥ 2).

Missing values of NSESSS scores per treatment arm were assessed; given its unbalanced distribution favoring the treated arms, we performed two sensitivity analyses by imputing either the best or the worst NSESSS value for each arm.

## Results

Table 3 shows the participants' demographics in the three randomized groups.

**Table 3:** Distributions of severity levels at T0 for PTSD symptoms in the three randomized groups (Control, Online intervention, In-person intervention)

Variables	Severity		No intervention (Control)	Online	In-person
NSESSS	< 2	Count	57	57	54
		% in arm	75%	76%	72.9%
NSESSS	≥ 2	Count	18	15	16
		% in arm	23.6%	20%	21.6%

*NSESSS* National Stressful Events Survey PTSD Short Scale (Kilpatrick et al., 2013)

One out of three participants, regardless of the group, reported severe PTSD symptoms. When exploring the longitudinal course of trauma-related symptoms over 12 months, we found a significant decrease in PTSD symptoms (*β* = − 3.67, *SE* = 0.51, *p* < 0.001).

**Fig. 2:**
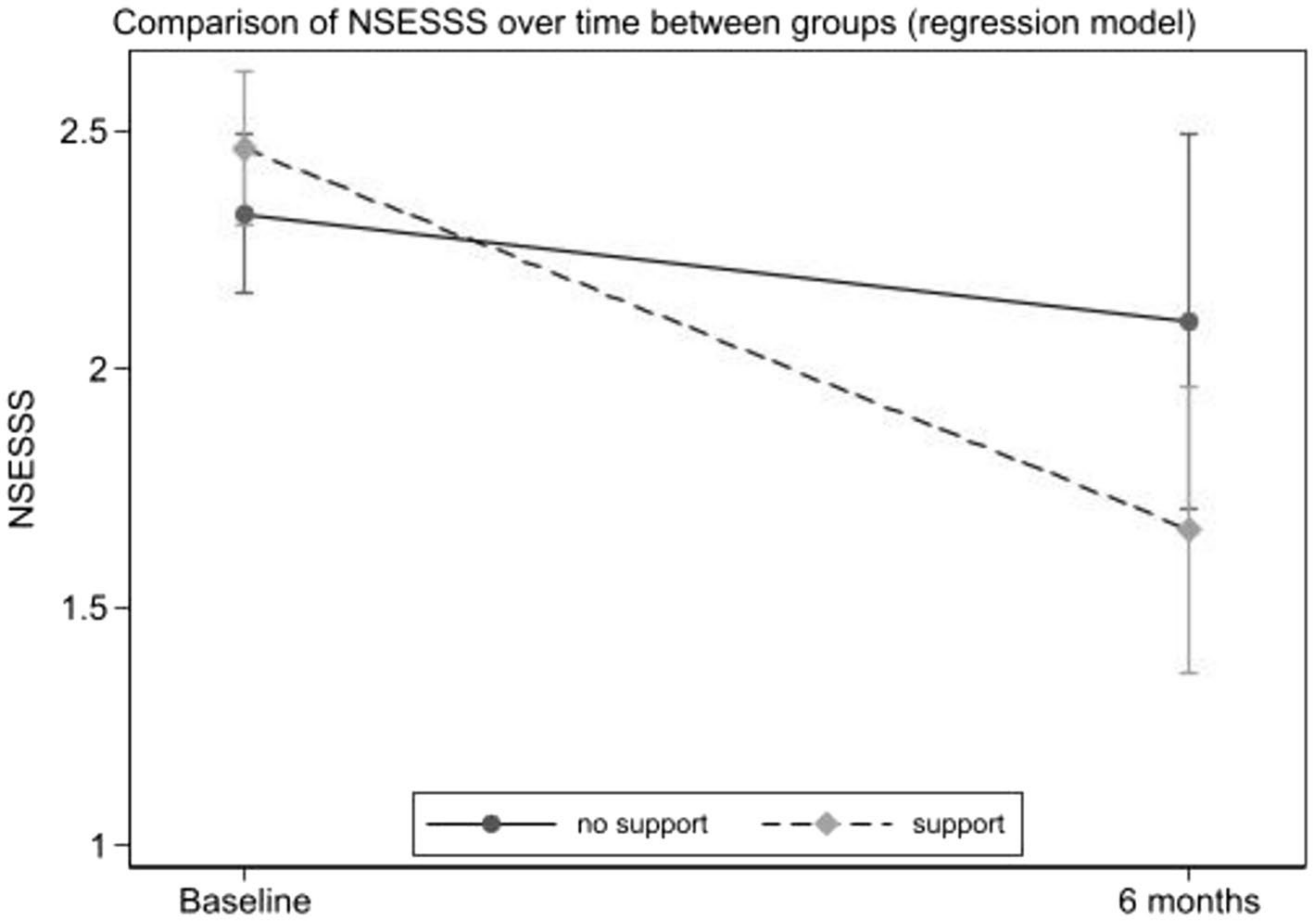
Changes over time of the NSESSS score in the Intervention and no Intervention arms. No support = no intervention arm; *Support* Intervention arm. *NSESS S* National Stressful Events Survey PTSD Short Scale (NSESSS) (Kilpatrick et al., 2013)

**Fig. 3:**
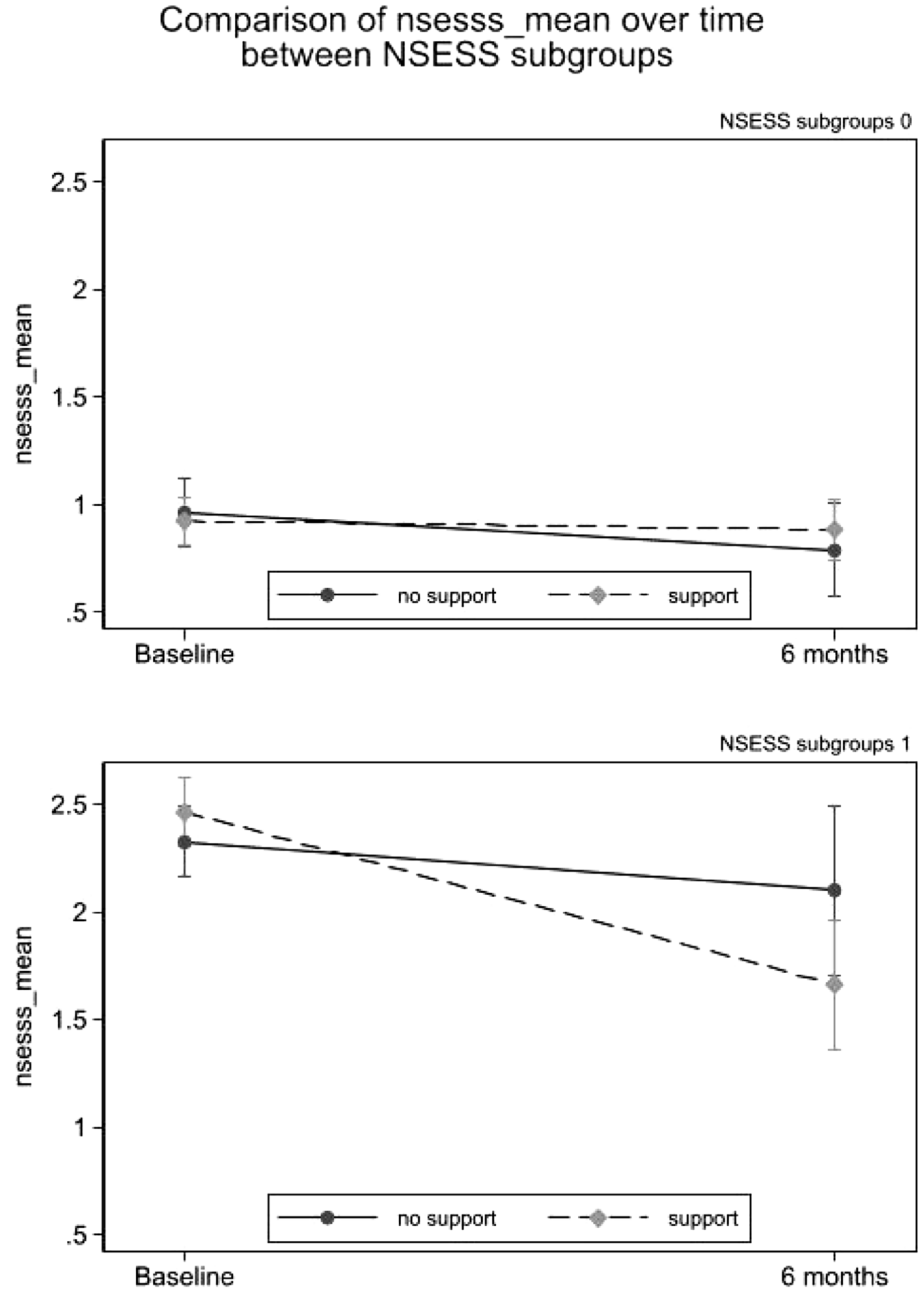
Subgroup analysis of the primary endpoint; left panel: subjects with NSESSS < 2 at baseline; right panel: subjects with NSESSS ≥ 2 at baseline. *No support* no intervention arm, *Support* Intervention arm. *NSESSS* National Stressful Events Survey PTSD Short Scale (NSESSS) (Kilpatrick et al., 2013)

When exploring the differences between groups (no intervention vs. intervention) in PTSD symptoms measured at six months and baseline, data showed no changes in PTSD symptoms after intervention (Fig. 2). However, results highlighted a significant decrease in the participants with severe PTSD symptoms participating in the psychological intervention compared to no intervention (Table 4; Fig. 3). No differences emerged between in-person and online intervention in these associations, *F*(2,47) = 0.265, *p* = 0.768. Moreover, data showed differences in this longitudinal course in gender, *F*(1,47) = 13.868, *p* < 0.001, but not in role, *F*(2,45) = 2.622, *p* = 0.073. Thus, females showed higher levels of PTSD symptoms compared to males. However, no interaction was found with the study condition, *F*(1,47) = 0.493, *p* = 0.484.

**Table 4:** Longitudinal differences (at T0 and T1) between Intervention and No Intervention groups in PTSD symptoms and subgroup analysis for severe PTSD symptoms

Variables	*N*	Intervention mean change from baseline (95% *CI*)	*N*	No intervention mean change from baseline (95% *CI*)	Mean difference between groups (95% *CI*)	*p* value
NSESSS	133	− 0.19 (− 0.34; − 0.05)	75	− 0.20 (− 0.38; − 0.02)	0.01 (− 0.22; 0.23)	0.948
Subgroup analysis						
Baseline NSESSS ≥ 2	31	− 0.80 (− 1.16; − 0.44)	18	− 0.23 (− 0.61; 0.16)	− 0.57 (− 1.08; − 0.06)	0.029
Baseline NSESSS < 2	102	− 0.04 (− 0.19; 0.11)	57	− 0.17 (− 0.37; 0.03)	0.14 (− 0.11; 0.38)	0.341
Sensitivity analysis*						
NSESSS best	149	− 0.31 (− 0.46; − 0.16)	76	− 0.22 (− 0.40; − 0.03)	− 0.09 (− 0.33;0.14)	0.434
NSESSS worst	149	0.02 (− 0.16; 0.19)	76	− 0.17 (− 0.34; 0.00)	0.19 (− 0.05; 0.43)	0.128

16 patients had missing values for NSESS at six months (6 online; 10 in-person) in the Intervention arm; 1 patient had a missing value for NSESSS in the control arm; the best and worse scores per arm are included in the sensitivity analysis

*NSESSS* National Stressful Events Survey PTSD Short Scale (Kilpatrick et al., 2013) *T0* before intervention, *T1* 6 months after intervention

## Discussion

The present study aimed to investigate how much trauma-related symptoms and behaviors exhibited by healthcare workers (HCWs) during the COVID-19 pandemic may change. Additionally, the study aimed to discern any effects in trauma-related symptoms and behaviors reduction associated with implementing a brief dialectical behavior therapy (DBT)-informed intervention. Considering the accumulating evidence on the psychological impact of COVID-19 on HCWs operating in hospital environments, our research aimed to contribute to this discourse by investigating an unresolved issue: namely, whether, how, and for whom the decrease in such impairment may be influenced solely by the passage of time, or whether the implementation of a psychological intervention could significantly impact it.

Initially, we aimed to chart the intra- and inter-individual longitudinal trajectories of trauma-related symptoms among frontline healthcare workers over 12 months. In alignment with our hypothesis, we observed a decrease in symptoms of Posttraumatic Stress Disorder (PTSD) over a year. This finding corroborates earlier studies that have suggested a natural decrease in PTSD symptoms over time, particularly in scenarios such as the current one, where behaviors and trauma-related symptoms are correlated with an event that progressively becomes more familiar and manageable in severity as time progresses (d'Ettorre et al., 2021; Negri et al., 2023).

Our secondary aim was to examine the intra-individual and inter-individual variations in trauma-related psychological functioning among healthcare workers (HCWs) by comparing two conditions through a randomized controlled trial (RCT) that manipulated the presence or absence of an intervention. The results indicated that participation in the proposed intervention did not significantly alter trauma-related behaviors and symptomatology for the group. In other words, whether HCWs participated in the intervention did not significantly influence their overall trauma-related functioning. However, an interesting distinction emerged when focusing on individuals most impacted by psychological trauma-related functioning. For these individuals, the intervention demonstrated effectiveness in mitigating their symptoms. This observation underscores the importance of not indiscriminately distributing psychological interventions to all HCWs, but rather adopting a more targeted and efficient approach (Hao et al., 2021). Also, it supports the usefulness of targeting emotion regulation to tackle these symptoms (Velotti et al., 2021). However, no differences emerged when considering online vs. in-person intervention, suggesting that both forms were equally effective in reducing PTSD symptoms. This finding adds to the recent literature on online psychological interventions that indicates the utility of providing online treatment when in-person is unavailable or difficult to administer (Jericho et al., 2022; Parolin et al., 2021; Tomaino et al., 2022).

Moreover, findings showed that females displayed higher trauma-related symptoms. This finding aligns with the literature suggesting a higher prevalence of PTSD symptoms in females (Carmassi et al., 2020). On the other hand, no differences emerged between different roles showing that trauma-related symptoms similarly impacted frontline HCWs. This finding might be explained by the fact that the study had considered only frontline workers and not compared them with HCWs involved in other units (Wu et al., 2009).

All in all, our findings advocate for a cost/benefits strategy that could prove highly advantageous by focusing on treating only those individuals experiencing more severe symptoms, as they stand to gain the most from mental health treatments. Furthermore, addressing the needs of these mainly affected HCWs in a cost/benefit balanced manner could be a productive strategy to achieve the broader objective of reducing the overall trauma-related psychological impairment within hospital units. The untreated psychological distress in this subset of professionals could have a ripple effect, potentially impacting the entire care system.

These results agree with previous research emphasizing the necessity for timely and targeted mental health interventions for frontline healthcare workers at a heightened risk of developing and sustaining trauma-related behaviors that could significantly compromise their professional caregiving capabilities.

All in all, the interpretations of this study's findings should be considered in light of its various limitations. First, we did not assess therapists’ adherence to the treatment: this might have led to differences in techniques and personal styles that might have resulted in discrepancies among them. Furthermore, given that the study intervention and sample differed from orthodox DBT and the typical DBT population, extant measures of DBT treatment fidelity were unlikely to be valid. Nevertheless, the treatment—including the DBT strategies in each session—was planned and taught by a DBT-certified trainer, who subsequently provided supervision throughout the treatment. Future studies should avoid this limitation, including assessing therapists’ techniques, style, and level of expertise. Second, the lack of difference in PTSD symptoms between those who received the intervention and those who did not might be well explained by the passage of time or the nature and duration of our DBT-informed intervention. In our study, participants underwent a brief intervention consisting of only five sessions, which contrasts the traditional tripartite DBT format that usually extends over a year and includes individual, group, and consultation sessions. Such a condensed format might have affected the depth and breadth of the therapeutic content, possibly rendering it insufficient to induce more substantial changes in the participants' PTSD symptoms.

Third, the results might be specific to our sample, and replication studies with diverse samples would bolster their generalizability. Fourth, our reliance on self-report measures may not have fully captured the complexities of the investigated variables. Future research could benefit from alternative methodologies, such as the experience sampling method, for a more detailed and dynamic understanding of individuals' daily experiences. Finally, the study was not pre-registered. As such, our approach has the potential for post hoc reasoning or unintended biases.

In conclusion, the present study provides valuable insights into the dynamics of trauma-related symptoms and behaviors exhibited by healthcare workers during the COVID-19 pandemic. Findings highlighted that these symptoms naturally decrease over time, particularly when associated with increasingly familiar and manageable events. Notably, this research also highlights the potential benefits of a brief DBT-informed intervention for those individuals most impacted by trauma-related psychological distress, suggesting that targeted interventions might be more effective than broad-based ones.

Our findings have significant implications for clinical treatment and intervention. Importantly, they suggest a nuanced approach to intervention deployment that does not uniformly target all healthcare professionals but prioritizes those with more severe symptoms. This approach might help maximize the cost/benefit ratio and mitigate the potential systemic impact of untreated severe psychological distress within healthcare settings.

## Data Availability

Data are available upon reasonable request.

## References

[CR1] Amerio, A., Brambilla, A., Morganti, A., Aguglia, A., Bianchi, D., Santi, F., Costantini, L., Odone, A., Costanza, A., & Signorelli, C. (2020). COVID-19 lockdown: Housing built environment’s effects on mental health. *International Journal of Environmental Research and Public Health,**17*(16), 5973. 32824594 10.3390/ijerph17165973 PMC7459481

[CR2] APA. (2013). Diagnostic and statistical manual of mental disorders—Fifth Edition. *Am Psychiatric Assoc,**21*(21), 591–643.

[CR3] Bersano, A., Kraemer, M., Touzé, E., Weber, R., Alamowitch, S., Sibon, I., & Pantoni, L. (2020). Stroke care during the COVID-19 pandemic: Experience from three large European countries. *European Journal of Neurology,**27*(9), 1794–1800. 32492764 10.1111/ene.14375 PMC7300856

[CR4] Bertuzzi, V., Semonella, M., Bruno, D., Manna, C., Edbrook-Childs, J., Giusti, E. M., Castelnuovo, G., & Pietrabissa, G. (2021). Psychological support interventions for healthcare providers and informal caregivers during the COVID-19 pandemic: A systematic review of the literature. *International Journal of Environmental Research and Public Health,**18*(13), 6939. 34203529 10.3390/ijerph18136939 PMC8297206

[CR5] Cai, H., Tu, B., Ma, J., Chen, L., Fu, L., Jiang, Y., & Zhuang, Q. (2020). Psychological impact and coping strategies of frontline medical staff in Hunan between January and March 2020 during the outbreak of coronavirus disease 2019 (COVID-19) in Hubei, China. *Medical Science Monitor: International Medical Journal of Experimental and Clinical Research,**26*, e924171–e924181. 32291383 10.12659/MSM.924171 PMC7177038

[CR6] Caramello, V., Gariglio, V., Di Salvo, G., Maina, G., & Boccuzzi, A. (2023). Longitudinal assessment of mental health consequences of the COVID-19 pandemic long-term exposure on health care workers from a North West Italian hospital. *Disaster Medicine and Public Health Preparedness,**17*, e378. 36891915 10.1017/dmp.2023.42 PMC10125871

[CR7] Carmassi, C., Foghi, C., Dell’Oste, V., Cordone, A., Bertelloni, C. A., Bui, E., & Dell’Osso, L. (2020). PTSD symptoms in healthcare workers facing the three coronavirus outbreaks: What can we expect after the COVID-19 pandemic. *Psychiatry Research,**292*, 113312. 32717711 10.1016/j.psychres.2020.113312 PMC7370915

[CR8] Chirico, F., Nucera, G., & Magnavita, N. (2020). COVID-19: Protecting healthcare workers is a priority. *Infection Control & Hospital Epidemiology,**41*(9), 1117–1117. 32299519 10.1017/ice.2020.148 PMC7198459

[CR9] d’Ettorre, G., Ceccarelli, G., Santinelli, L., Vassalini, P., Innocenti, G. P., Alessandri, F., Koukopoulos, A. E., Russo, A., d’Ettorre, G., & Tarsitani, L. (2021). Posttraumatic stress symptoms in healthcare workers dealing with the COVID-19 pandemic: A systematic review. *International Journal of Environmental Research and Public Health,**18*(2), 601. 33445712 10.3390/ijerph18020601 PMC7828167

[CR10] De Kock, J. H., Latham, H. A., Leslie, S. J., Grindle, M., Munoz, S.-A., Ellis, L., Polson, R., & O’Malley, C. M. (2021). A rapid review of the impact of COVID-19 on healthcare workers’ mental health: Implications for supporting psychological well-being. *BMC Public Health,**21*(1), 1–18. 33422039 10.1186/s12889-020-10070-3 PMC7794640

[CR11] Galli, F., Pozzi, G., Ruggiero, F., Mameli, F., Cavicchioli, M., Barbieri, S., Canevini, M. P., Priori, A., Pravettoni, G., & Sani, G. (2020). A systematic review and provisional metanalysis on psychopathologic burden on health care workers of coronavirus outbreaks. *Frontiers in Psychiatry,**11*, 568664. 33192692 10.3389/fpsyt.2020.568664 PMC7596413

[CR12] Giusti, E. M., Pedroli, E., D’Aniello, G. E., Stramba Badiale, C., Pietrabissa, G., Manna, C., Stramba Badiale, M., Riva, G., Castelnuovo, G., & Molinari, E. (2020). The psychological impact of the COVID-19 outbreak on health professionals: A cross-sectional study. *Frontiers in Psychology,**11*, 1684. 32754102 10.3389/fpsyg.2020.01684 PMC7366071

[CR13] Greenberg, N., Weston, D., Hall, C., Caulfield, T., Williamson, V., & Fong, K. (2021). Mental health of staff working in intensive care during Covid-19. *Occupational Medicine,**71*(2), 62–67. 33434920 10.1093/occmed/kqaa220 PMC7928568

[CR14] Hao, Q., Wang, D., Xie, M., Tang, Y., Dou, Y., Zhu, L., Wu, Y., Dai, M., Wu, H., & Wang, Q. (2021). Prevalence and risk factors of mental health problems among healthcare workers during the COVID-19 pandemic: A systematic review and meta-analysis. *Frontiers in Psychiatry,**12*, 567381. 34211406 10.3389/fpsyt.2021.567381 PMC8239157

[CR15] Harvey, L. J., Hunt, C., & White, F. A. (2019). Dialectical behaviour therapy for emotion regulation difficulties: A systematic review. *Behaviour Change,**36*(3), 143–164.

[CR16] Jericho, B., Luo, A., & Berle, D. (2022). Trauma-focused psychotherapies for posttraumatic stress disorder: A systematic review and network meta-analysis. *Acta Psychiatrica Scandinavica,**145*(2), 132–155. 34473342 10.1111/acps.13366 PMC9539869

[CR17] Kilpatrick, D. G., Resnick, H. S., & Friedman, M. J. (2013). *Severity of acute stress symptoms—Adult (national stressful events survey acute stress disorder short scale [NSESSS])*. American Psychiatric Association.

[CR18] Lai, J., Ma, S., Wang, Y., Cai, Z., Hu, J., Wei, N., Wu, J., Du, H., Chen, T., & Li, R. (2020). Factors associated with mental health outcomes among health care workers exposed to coronavirus disease 2019. *JAMA Network Open,**3*(3), e203976–e203976. 32202646 10.1001/jamanetworkopen.2020.3976 PMC7090843

[CR19] Lee, A. M., Wong, J. G., McAlonan, G. M., Cheung, V., Cheung, C., Sham, P. C., Chu, C.-M., Wong, P.-C., Tsang, K. W., & Chua, S. E. (2007). Stress and psychological distress among SARS survivors 1 year after the outbreak. *The Canadian Journal of Psychiatry,**52*(4), 233–240. 17500304 10.1177/070674370705200405

[CR20] Linehan, M. M. (1993). *Skills training manual for treating borderline personality disorder*. Guilford press.

[CR21] Linehan, M. M., & Wilks, C. R. (2015). The course and evolution of dialectical behavior therapy. *American Journal of Psychotherapy,**69*(2), 97–110. 26160617 10.1176/appi.psychotherapy.2015.69.2.97

[CR22] Luo, M., Guo, L., Yu, M., Jiang, W., & Wang, H. (2020). The psychological and mental impact of coronavirus disease 2019 (COVID-19) on medical staff and general public—A systematic review and meta-analysis. *Psychiatry Research,**291*, 113190. 32563745 10.1016/j.psychres.2020.113190 PMC7276119

[CR23] Marcon, E., Scotton, F., Marcante, E., Rigo, A., Monticelli, J., Buggio, M. E., Pilerci, C., Montemurro, D., & Benini, P. (2020). Schiavonia hospital response to COVID-19 outbreak: A first single-center experience. *Annali Dell’istituto Superiore Di Sanita,**56*(3), 365–372. 32959803 10.4415/ANN_20_03_15

[CR24] McCoy, C. E. (2017). Understanding the Intention-to-treat principle in randomized controlled trials. *Western Journal of Emergency Medicine,**18*(6), 1075. 10.5811/westjem.2017.8.35985 29085540 10.5811/westjem.2017.8.35985 PMC5654877

[CR25] Mosheva, M., Gross, R., Hertz-Palmor, N., Hasson-Ohayon, I., Kaplan, R., Cleper, R., Kreiss, Y., Gothelf, D., & Pessach, I. M. (2021). The association between witnessing patient death and mental health outcomes in frontline COVID-19 healthcare workers. *Depression and Anxiety,**38*(4), 468–479. 33544405 10.1002/da.23140 PMC8014064

[CR26] Neacsiu, A. D., Eberle, J. W., Kramer, R., Wiesmann, T., & Linehan, M. M. (2014). Dialectical behavior therapy skills for transdiagnostic emotion dysregulation: A pilot randomized controlled trial. *Behaviour Research and Therapy,**59*, 40–51. 24974307 10.1016/j.brat.2014.05.005

[CR27] Negri, L., Bassi, M., Accardi, R., & DelleFave, A. (2023). Posttraumatic stress symptoms and benefit finding: a longitudinal study among Italian health workers during the COVID-19 pandemic. *Social Psychiatry and Psychiatric Epidemiology*. 10.1007/s00127-023-02475-3 37029827 10.1007/s00127-023-02475-3 PMC10082687

[CR28] Orrù, G., Ciacchini, R., Gemignani, A., & Conversano, C. (2020). Psychological intervention measures during the COVID-19 pandemic. *Clinical Neuropsychiatry,**17*(2), 76. 34908972 10.36131/CN20200208 PMC8629089

[CR29] Pappa, S., Ntella, V., Giannakas, T., Giannakoulis, V. G., Papoutsi, E., & Katsaounou, P. (2020). Prevalence of depression, anxiety, and insomnia among healthcare workers during the COVID-19 pandemic: A systematic review and meta-analysis. *Brain, Behavior, and Immunity,**88*, 901–907. 32437915 10.1016/j.bbi.2020.05.026 PMC7206431

[CR30] Parolin, L. A. L., Benzi, I. M. A., Fanti, E., Milesi, A., Cipresso, P., & Preti, E. (2021). Italia Ti Ascolto [Italy, I am listening]: An app-based group psychological intervention during the COVID-19 pandemic. *Research in Psychotherapy: Psychopathology, Process, and Outcome,**24*(1), 517. 33937116 10.4081/ripppo.2021.517 PMC8082536

[CR31] Rizzi, D., Asperges, E., Rovati, A., Bigoni, F., Pistillo, E., Corsico, A., Mojoli, F., Perlini, S., & Bruno, R. (2022). Psychological support in a COVID-19 hospital: A community case study. *Frontiers in Psychology,**12*, 6699. 10.3389/fpsyg.2021.820074 PMC8893142 35250697

[CR32] Robinson, E., Sutin, A. R., Daly, M., & Jones, A. (2022). A systematic review and meta-analysis of longitudinal cohort studies comparing mental health before versus during the COVID-19 pandemic in 2020. *Journal of Affective Disorders,**296*, 567–576. 34600966 10.1016/j.jad.2021.09.098 PMC8578001

[CR33] Ruiz-Fernández, M. D., Ramos-Pichardo, J. D., Ibáñez-Masero, O., Cabrera-Troya, J., Carmona-Rega, M. I., & Ortega-Galán, Á. M. (2020). Compassion fatigue, burnout, compassion satisfaction and perceived stress in healthcare professionals during the COVID-19 health crisis in Spain. *Journal of Clinical Nursing,**29*(21–22), 4321–4330. 32860287 10.1111/jocn.15469

[CR34] Storebø, O. J., Stoffers-Winterling, J. M., Völlm, B. A., Kongerslev, M. T., Mattivi, J. T., Jørgensen, M. S., Faltinsen, E., Todorovac, A., Sales, C. P., & Callesen, H. E. (2020). Psychological therapies for people with borderline personality disorder. *Cochrane Database of Systematic Reviews*. 10.1002/2F14651858.CD012955.pub2 32368793 10.1002/14651858.CD012955.pub2 PMC7199382

[CR35] Taylor, S. (2019). *The psychology of pandemics: Preparing for the next global outbreak of infectious disease*. Cambridge Scholars Publishing.

[CR36] Tomaino, S. C. M., Viganò, G., & Cipolletta, S. (2022). The COVID-19 crisis as an evolutionary catalyst of online psychological interventions. A systematic review and qualitative synthesis. *International Journal of Human-Computer Interaction*. 10.1080/10447318.2022.2111047

[CR37] Varela, C., Montero, M., Serrano-Ibáñez, E. R., de la Vega, A., & Pulido, M. A. G. (2023). Psychological interventions for healthcare professionals during the COVID-19 pandemic: A systematic review. *Stress and Health*. 10.1002/smi.3246 37052296 10.1002/smi.3246

[CR38] Velotti, Patrizia, Civilla, Claudia, Rogier, Guyonne, & Zobel, Sara Beomonte. (2021). A fear of COVID-19 and PTSD symptoms in pathological personality: the mediating effect of dissociation and emotion dysregulation. *Frontiers in psychiatry,**12*, 590021. 33833698 10.3389/fpsyt.2021.590021 PMC8021772

[CR39] Vizheh, M., Qorbani, M., Arzaghi, S. M., Muhidin, S., Javanmard, Z., & Esmaeili, M. (2020). The mental health of healthcare workers in the COVID-19 pandemic: A systematic review. *Journal of Diabetes & Metabolic Disorders,**19*, 1967–1978. 33134211 10.1007/s40200-020-00643-9 PMC7586202

[CR40] Walton, M., Murray, E., & Christian, M. D. (2020). Mental health care for medical staff and affiliated healthcare workers during the COVID-19 pandemic. *European Heart Journal: Acute Cardiovascular Care,**9*(3), 241–247. 32342698 10.1177/2048872620922795 PMC7189614

[CR41] Wu, P., Fang, Y., Guan, Z., Fan, B., Kong, J., Yao, Z., Liu, X., Fuller, C. J., Susser, E., & Lu, J. (2009). The psychological impact of the SARS epidemic on hospital employees in China: Exposure, risk perception, and altruistic acceptance of risk. *The Canadian Journal of Psychiatry,**54*(5), 302–311. 19497162 10.1177/070674370905400504 PMC3780353

